# Supplementary material for: Effects of Lumacaftor-Ivacaftor on Airway Microbiota-Mycobiota and Inflammation in Patients with Cystic Fibrosis Appear To Be Linked to Pseudomonas aeruginosa Chronic Colonization
Source: Microbiol Spectr. 2023 Mar 27;11(2):e02251-22. doi: 10.1128/spectrum.02251-22 (PMC10100832; doi:10.1128/spectrum.02251-22)
Supplement: Supplemental file 1 — Supplemental material. Download spectrum.02251-22-s0001.pdf, PDF file, 0.9 MB [file spectrum.02251-22-s0001.pdf]

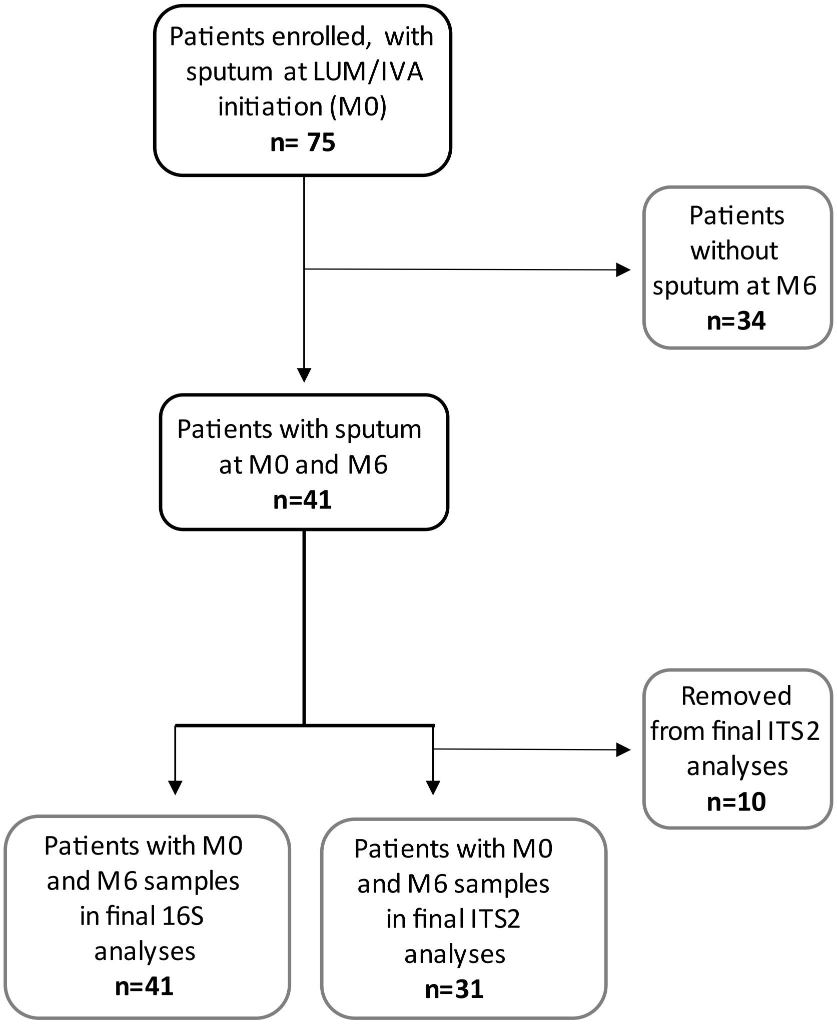


**Figure S1. Flowchart of Lum-Iva-Biota study and samples distribution across time**

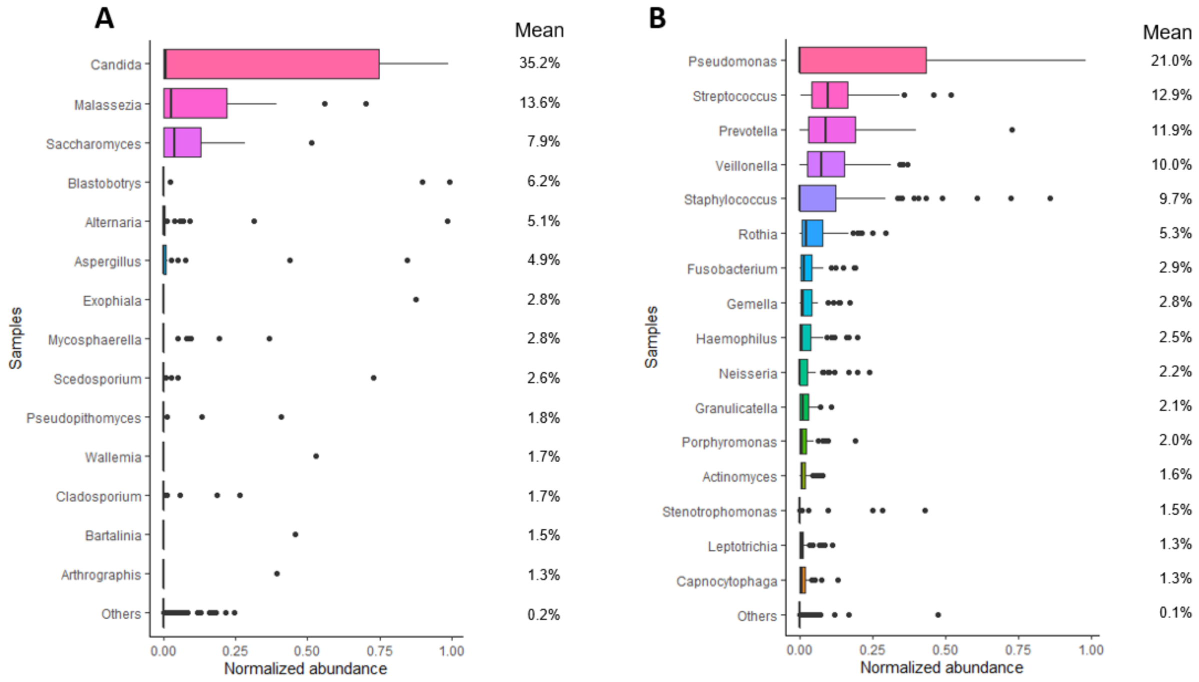


**Figure S2. Fungal (A) and bacterial (B) genera that composed sputum microbial communities of CF patients (n=75) at baseline (M0)**

At baseline, fungal genera were dominated by *Candida* (35%), followed by *Malassezia* (14%) and *Saccharomyces* (8%). *Aspergillus* represented 5% of the fungal ASVs (**A**). Bacterial genera were dominated by *Pseudomonas* (21%), followed by *Streptococcus* (13%), and *Prevotella* (12%). *Veillonella* and *Staphylococcus* each represented 10% of the bacterial ASVs (**B**).

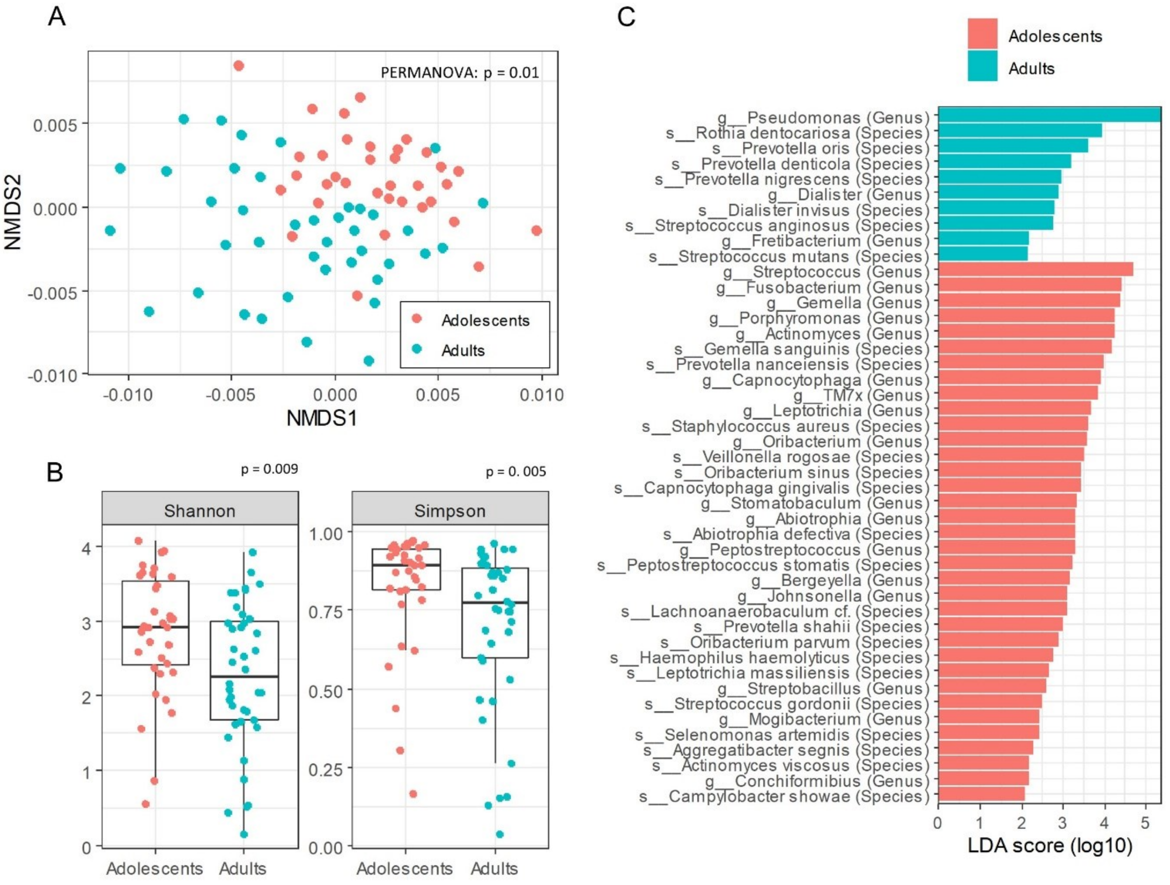


**Figure S3. Bacterial composition of sputum at baseline according to age**

Beta diversity which assesses differences in microbiota (A) composition between samples using a Non-metric multidimensional scaling (NMDS) ordination method with Bray–Curtis distance metric. Alpha diversity indices (Observed ASVs. Shannon and Simpson) for microbiota (B). LEfSe analysis showing ASVs distinguishing adolescent from adult airway microbiota at baseline (p-value < 0.05) (C).

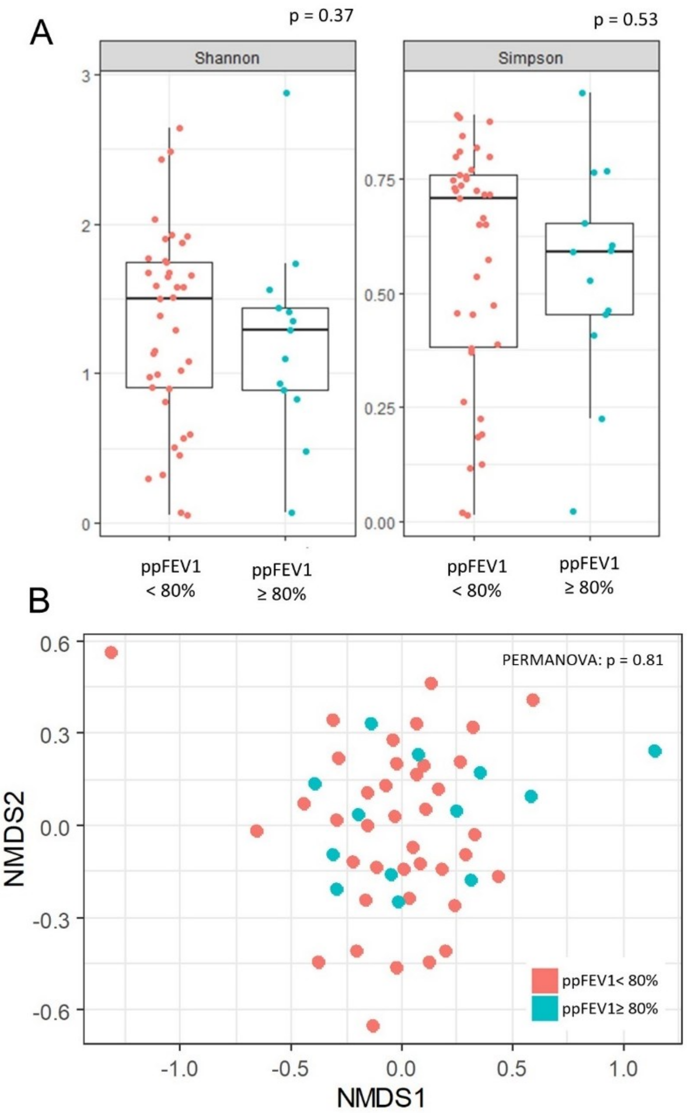


**Figure S4. Fungal composition of sputum at baseline according to lung function**

Alpha-diversity indices (Shannon and Simpson) for mycobiota (**A**). Beta-diversity which assesses differences in mycobiote (**B**) composition between samples using a Non-Metric MultiDimensional Scaling (NMDS) ordination method with Bray–Curtis distance metric.

**Table S1. Clinical characteristics at baseline according to *Pseudomonas aeruginosa* colonization**

| | **Missing values** | ***P. aeruginosa* colonized patients at baseline n=41 (53%)** | **Patients not chronically colonized with *P. aeruginosa* at baseline n=34 (47%)** | **p-value** |
|---|---|---|---|---|
| **Age (Years)** | 0 | 25.8 (±9.11)** | 15.1 (±4.09) | **<0.001** |
| **Gender (Female)** | 0 | 22 (54%) | 18 (53%) | 0.95 |
| **$ppFEV_1$*** | 4 | 56.0 [41.0; 69.0] | 79.0 [63.2; 92.2] | **<0.001** |
| **BMI‡, kg/$m^2$** | 2 | 19.2 [18.2; 22.0] | 18.3 [16.8; 20.2] | **0.05** |
| **BMI, Z-Score‡‡** | | -0.96 [-1.31; -0.60] | -0.60 [-1.08; 0.24] | 0.093 |
| **≥1 IV antibiotic courses in the previous 12 months#** | 4 | 11 (28%) | 4 (12%) | **<0.01** |
| **Maintenance pulmonary medications at baseline** | | | | |
| Inhaled antibiotics | 1 | 33 (82%) | 12 (35%) | **<0.001** |
| Azithromycin | 0 | 31 (76%) | 14 (41%) | **<0.01** |
| Dornase alfa | 1 | 29 (72%) | 30 (88%) | 0.09 |
| Inhaled corticosteroids | 0 | 30 (73%) | 22 (65%) | 0.43 |
| Oral corticosteroids | 0 | 2 (4.9%) | 1 (2.9%) | 1 |
| Inhaled hypertonic saline | 5 | 2 (5.1%) | 6 (19%) | 0.13 |
| Inhaled bronchodilators | 5 | 32 (82%) | 27 (87%) | 0.74 |
| **Pulmonary colonization** | | | | |
| MSSA†† | 0 | 25 (61%) | 20 (59%) | 0.85 |
| MRSA††† | 0 | 8 (20%) | 4 (12%) | 0.36 |
| *H. influenzae* | 0 | 3 (7.3%) | 4 (12%) | 0.69 |
| *B. cepacia* | 0 | 2 (4.9%) | 0 (0%) | 0.5 |
| *A. fumigatus*†††† | 16 | 13 (42%) | 10 (36%) | 0.62 |
| **Sputum supernatant dosages** | | | | |
| Calprotectin (µg/mL) | 1 | 4278 [3827; 4743] | 3168 [768; 3902] | **<0.001** |
| Patient with GM index > 1 | 5 | 14 (35%) | 5 (17%) | 0.08 |
| Total fungal load (log pg/µL) | 0 | 0.7 [0; 1.3] | 0.7 [0; 1.1] | 0.7 |
| Total bacterial load (log pg/µL) | 0 | 2. [1.7; 2.7] | 2.4 [2.0; 3.0] | 0.2 |
| *P. aeruginosa* (log copies/mL) | 1 | 6.1 [3.7; 7.3] | 2.4 [2.2; 2.7] | **<0.001** |

*ppFEV1: percent predicted forced expiratory volume in 1 sec ; **Data are mean (±SD), median [IQR] or n (%); ‡BMI: body mass index; ‡‡For adolescents, #These criteria were evaluated during the year previous of the visit; ††MSSA : methicillin-susceptible *S. aureus* ; †††MRSA: methicillin-resistant *S. aureus. †††† A. fumigatus colonization referred to chronic colonization status defined as 2 sputum cultures positive for A. fumigatus during the last 12 months (31).*

**Table S2. Significant changes in bacterial and fungal composition of sputum after 6 months of lumacaftor-ivacaftor treatment in patients not chronically colonized with *P. aeruginosa at baseline***

| Kingdom | Phylum | Order | Family | Genus | Species | Gram | log2Fold Change | lfcSE | p adjuted |
|---|---|---|---|---|---|---|---|---|---|
| Fungi | Basidiomycota | Malasseziales | Malasseziaceae | *Malassezia* | *restricta* | - | 24.52 | 6.62 | 0.03 |
| Bacteria | Actinobacteriota | Micrococcales | Micrococcaceae | *Rothia* | *mucilaginosa* | Positive | -20.83 | 5.52 | 0.04 |
| Bacteria | Patescibacteria | Saccharimonadales | Saccharimonadaceae | TM7x | | Positive | -21.27 | 5.34 | 0.03 |
| Bacteria | Firmicutes | Veillonellales | Veillonellaceae | *Veillonella* | | Negative | -21.81 | 5.56 | 0.03 |
| Bacteria | Bacteroidota | Flavobacteriales | Flavobacteriaceae | *Capnocytophaga* | *sputigena* | Negative | -22.50 | 6.02 | 0.04 |
| Fungi | Ascomycota | Saccharomycetales | Saccharomycetales | *Candida* | *albicans* | - | -22.97 | 6.66 | 0.04 |
| Bacteria | Fusobacteriota | Fusobacteriales | Fusobacteriaceae | *Fusobacterium* | *periodonticum* | Negative | -24.26 | 5.47 | 0.01 |

**Supplementary material**

In house artificial bacterial and fungal communities were composed of:

- *Streptococcus mitis*, *Streptococcus oralis*, *Pseudomonas aeruginosa*, *Stenotrophomonas maltophilia*, *Staphylococcus epidermidis*, *Staphylococcus aureus*, *Acinetobacter baumannii*, *Klebsiella pneumoniae*, *Proteus mirabilis*, *Serratia marcescens*, *Lactobacillus sp.*, *Enterobacter cloacae* (all corresponding to patient isolates kindly provided by Pr. P Lehours and Pr. S. Pereyre, Bacteriology lab of Bordeaux CHU), *Escherichia coli* (ATCC 25922), and *Enterococcus faecalis* (ATCC 29212), and
- *Scedosporium apiospermum*, *Scedosporium aurantiacum*, *Lomentospora prolificans (previously named Scedosporium prolificans)*, *Mucor circillenoide*, *Aspergillus flavus*, *Aspergillus terreus*, *Penicillium griseofulvum*, *Fusarium solani*, *Exophiala dermatitidis*, *Rhodotorula mucilaginosa*, *Candida dubliniensis*, *Candida lusitaniae*, (all corresponding to patient isolates provided by Pr. L. Delhaes, Mycology lab of Bordeaux CHU), and *Candida albicans* (ATCC 5314), and *Aspergillus fumigatus (Strain DAL)*.
